# Effect of Occupational Stress on Periodontitis According to the Salivary RANKL Level Among Iraqi Employees

**DOI:** 10.2147/CCIDE.S455831

**Published:** 2024-03-12

**Authors:** Athraa Ali Mahmood, Hussain Owaid Muhammed Al-Obadi, Hashim Mueen Hussein

**Affiliations:** 1 Department of Oral Surgery and Periodontics, College of Dentistry, Mustansiriyah University, Baghdad, Iraq; 2 Department of Conservative Dentistry, College of Dentistry, Mustansiriyah University, Baghdad, Iraq

**Keywords:** periodontitis, occupational stress, salivary RANKL, employees

## Abstract

**Background:**

Findings show that periodontitis does not affect all populations; similarly, some individuals present risk conditions such as occupational stress, making them more susceptible to developing periodontitis through unhealthy habits like poor oral hygiene and immune suppression. Periodontitis triggers an inflammatory host immune response; “Receptor Activator Nuclear Factor KB ligand (RANKL)” is the primary regulator of osteoclast differentiation and activity. It was found that osteoclastic bone damage caused by periodontitis depends on the RANKL produced by osteoblastic and periodontal ligament cells.

**Objective:**

This study aimed to assess the effect of occupational stress on employees with periodontitis using salivary RANKL marker.

**Material and Methods:**

A case–control analysis was done at my clinic with 90 male employees aged 30–50. The participants completed self-administered questionnaires and had periodontal exams. Employee occupational stress was estimated using a life events scale questionnaire. Calibrated dentists performed the parameters used in the periodontal assessment after collecting whole unstimulated salivary samples from each employee to measure salivary RANKL using ELISA technique.

**Results:**

The present finding revealed a statistically significant difference among groups in “probing pocket depth, plaque index, bleeding on probing, clinical attachment level, and salivary RANKL level”. They were higher in the stressed employees’ group, which is not statistically significant.

**Conclusion:**

The findings of this investigation observed that occupational stress increased clinical periodontal parameters and salivary RANKL of periodontitis in employees.

## Introduction

Periodontitis is a destructive inflammatory disease that can cause loss of attachment apparatus, alveolar bone, and pathological pockets around the teeth.^1–3^ Several studies findings have found that periodontitis does not influence all populations similarly; some people have risk conditions or factors that make them susceptible to developing periodontal diseases, such as the virulence of the microorganism, immunological, and genetic mechanisms, and host environmental conditions, mainly smoking, psychosocial stress, and lifestyle factors.^4–11^

Stress is a psychological and physiological response effect of environmental variation and noxious stimulation, leading to disease if extensive.^12^ Stress is not harmful if a person has active coping behaviours and can control his environment.^4^

Several studies have linked stress factors to periodontal disease. Stress can indirectly affect the health of periodontal status by activating harmful factors like poor oral hygiene, an imbalanced diet, heavy smoking, and more doses of drugs or alcohol; In addition, periodontal health can be affected directly through a biological source that occurs by changes in the saliva amount and constituents, and changes in the gingival blood circulation that can be regulated by the immune system of the patient itself.^13–16^

Work-related stress, “Occupational stress”, has been reported as a predisposing factor to periodontal disease in staff working.^17–19^ It’s becoming more serious globally due to job involvement, notable changes in the working period, and workplace environment.^20^,^21^ Thus, “World Health Organization” defines occupational stress as a worldwide pandemic and is studying its severity.^22^ It adversely affects employment and contributes to physical and mental diseases.^23^ For example, studies show that occupational stress is linked to specific oral health diseases like caries,^24^ periodontal disease,^16^,^25^ temporomandibular disorder, and halitosis.^26^ This association is biologically plausible with studies demonstrating that exposure to stress agents with inadequate coping could modify the immune response by acting on the central nervous system to release immunoregulatory neurotransmitters, neuropeptides, inflammatory cytokines, and glucocorticoid hormones such as cortisol.

Cortisol upregulates “Receptor Activator Nuclear Factor KB ligand (RANKL)” expressions in osteoblasts and stimulates osteoclast differentiation, activation, and survival; thus, negative bone turnover and loss may result through increased bone resorption, making the person more susceptible to developing disease and affecting periodontal health. So, the RANKL can play an essential work in periodontal destruction, while the RANKL inhibition can stop the destruction of the periodontium of the patients.^13^,^19^,^27^,^28^

As far as we know, no study has investigated the effect of occupational stress among employees on periodontal clinical parameters of periodontitis compared to healthy control employees according to the salivary RANKL level. Thus, the rationale of this study was to assess these effects among Iraqi employees.

## Materials and Methods

### Study Population

The study design was observational case-control conducted at a private clinic from March 2023 to September 2023. The study population included 90 male employees aged 30 to 50 years with the presence of ≥ twenty teeth. They were categorized into three groups: healthy periodontium “probing pocket depth (PPD) ≤3 mm without clinical attachment loss (CAL) and bleeding on probing (BOP) <10%” non-stressed control (30 subjects), periodontitis “(PPD) ≥ 5 mm or PPD ≥ 4 mm with BOP” non-stressed (30 patients), and periodontitis with occupational stress (30 patients).

The Mustansiriyah University College of Dentistry ethics committee approved this study. Our study excluded smokers, alcohol drinkers, obese, diabetics, immune system disorders, antibiotics, anti-inflammatory, immunosuppressive drug users, and unemployed participants.

### Procedure

Participants signed a written consent form that explained the study’s purpose. Then, each participant was asked to answer self-administered questions about the subject’s name, age, employment status, and information about dental care. Occupational stress was estimated using “a life events scale questionnaire”.^13^,^29^,^30^ Participants answered “yes” or “no” to agree or disagree with a statement. All procedures in this study followed ethical principles, including the ‘World Medical Association Declaration of Helsinki’ and its later amendments for human research.

After collecting whole unstimulated salivary samples from each participant, periodontal examination “plaque index (PLI), BOP, PPD, and CAL” were assessed, as the following: The percentage of surfaces with visible plaque after staining with disclosing agents was measured at each tooth’s mesial, distal, lingual, and facial aspects to assess dental plaque (occlusal surfaces being excluded). Then, BOP was measured immediately after PPD. A site was scored positively if bleeding occurred within 30 seconds of probing and negatively if not. They calculated the bleeding percentage of total surfaces.

In addition, sites with PPD ≥ 4mm were measured using a “Williams probe” inserted into the gingival crevice near the tooth’s long axis, counting from the gingival margin to the apex in millimeters. The measurement sites were mid-facial, mid-lingual, mesiofacial, distofacial, mesiolingual, and distolingual. The probe fell by its weight without pressure. It was measured to the millimetre. CAL represents the millimeter vertical distance from the cementoenamel junction to the probable gingival pocket bottom. The third molar was not recorded.

An enzyme-linked immunosorbent assay (ELISA) was used to detect the levels of RANKL in the collected salivary samples in this study.

### Statistical Analysis

Database (Excel for Windows) stored data and the results were subjected to statistical analysis using the software SPSS for Windows version 28.00. The histogram, quantile–quantile plot, and the Shapiro–Wilk test were used to identify the normality of the data distribution.

For the descriptive analysis, the mean, standard Deviation (SD) and median values of PLI, BOP, PPD, CAL, and salivary RANKL were calculated for each subject and compared among groups by Kruskal–Wallis H. In addition, it was compared for each group using a Mann–Whitney *U*-test; Spearman’s coefficient correlation determined the statistically significant correlations between clinical periodontal parameters and RANKL salivary level among employee groups. The comparison was 5% significant.

## Results

The sample included 90 employees who completed the periodontal clinical examination and a self-reported questionnaire. Sixty employees had periodontitis with and without occupational stress groups were regarded as (cases groups), while thirty were regarded as healthy-periodontium (control group) without occupational stress.

(Table 1) shows the total mean PLI percentage in the periodontitis non-stressed and stressed groups were 57.334% and 69.157%, respectively, while it was 10.424% for the control group, with a significant difference (P < 0.001), as illustrated in (Table 2).

**Table 1:** The Mean of PLI, BOP, PD, and CAL for All Sites and the Mean of Salivary RANKL

Parameter		Groups (n=90 employees)		
		(C) group (n=30)	(PNS) group (n=30)	(PS) group (n=30)
Age (years)	Mean±SD	37.066±6.096	39.900±7.958	43.266±8.362
	Median	35	38	44
PLI (%)	Mean±SD	10.424±4.481	57.334±22.224	69.157±21.496
	Median	10.180	64	78.015
BOP (%)	Mean±SD	3.969±2.985	41.638±18.418	52.062±16.162
	Median	3.290	38.800	51.500
PPD (mm)	Mean±SD	0.00±0.00	4.4520±0.63158	4.7953±.61054
	Median	0.00	4.2250	4.8450
CAL (mm)	Mean±SD	0.00±0.00	3.4487±1.02605	3.461±0.998
	Median	0.00	3.260	3.400
RANKL (ng/L)	Mean±SD	26.082±5.680	38.399±6.230	45.212±8.357
	Median	25.274	38.326	45.595

**Abbreviations**: C, Control non-stressed; PNS, Periodontitis non-stressed; PS, Periodontitis with stress SD, Standard Deviation; “PLI, Plaque index; BOP, Bleeding on probing; PPD, Probing pocket depth; CAL, Clinical attachment loss; RANKL, Receptor Activator Nuclear Factor KB ligand”; %, Percent; mm, Millimeter, ng/L, Nanogram per liter.

**Table 2:** Kruskal–Wallis H Revealed Significant Differences in the Groups’ Periodontal Parameters and the Salivary RANKL Levels. While “Mann–Whitney *U*-Test” Showed Significant and Non-Significant Differences Between Each Group

Parameter	Group	MeanRank	Kruskal–Wallis		Comparison	Mann–Whitney	
			value	P-value		value	P-value
Age (years)	C	36.32	7.989	0.018*	C vs PNS	363.500	0.199^NS^
	PNS	44.88			C vs PS	261.000	0.005**
	PS	55.30			PNS vs PS	345.000	0.119^NS^
PLI (%)	C	17.10	56.428	0.000***	C vs PNS	48.000	0.000***
	PNS	53.63			C vs PS	0.000	0.000***
	PS	65.77			PNS vs PS	292.000	0.019*
BOP (%)	C	15.50	61.588	0.000***	C vs PNS	0.000	0.000***
	PNS	55.47			C vs PS	0.000	0.000***
	PS	65.53			PNS vs PS	299.000	0.026*
PPD (mm)	C	15.50	64.958	0.000***	C vs PNS	0.000	0.000***
	PNS	55.08			C vs PS	0.000	0.000***
	PS	65.92			PNS vs PS	287.500	0.015*
CAL (mm)	C	15.50	61.636	0.000***	C vs PNS	0.000	0.000***
	PNS	60.18			C vs PS	0.000	0.000***
	PS	60.82			PNS vs PS	440.500	0.888^NS^
RANKL (ng/L)	C	18.40	53.708	0.000***	C vs PNS	65.000	0.000***
	PNS	51.30			C vs PS	22.000	0.000***
	PS	66.80			PNS vs PS	239.000	0.002**

**Notes**: significance level, *P< 0.05 **P≤ 0.01: ***P ≤ 0.001.

**Abbreviations**: C, Control non-stressed; PNS, Periodontitis non-stressed; PS, Periodontitis with stress; NS, Non-significant at P ≥ 0.05; “PLI, plaque index; BOP, bleeding on probing; PPD, probing pocket depth; CAL, clinical attachment loss; RANKL, Receptor Activator Nuclear Factor KB ligand ”; %, Percent; mm, Millimeter; ng/L, Nanogram per liter.

In addition, Table 1 describes the distribution of BOP percentage in each group. The mean percentages of probing site bleeding in the periodontitis non-stressed and stressed groups were 41.638% and 52.062%, respectively. In comparison, it is 3.969% for the control group, with a significant difference (P < 0.001), as illustrated in (Table 2).

We calculated the mean of sites with a PPD ≥ 4mm. Table (1) indicates a mean PPD rate of ≥ 4mm in the periodontitis non-stressed and stressed groups was 4.452mm and 4.795mm, respectively, while there was no pocket in the control group with significant difference (P < 0.001), as illustrated in (Table 2).

The total mean CAL for the periodontitis non-stressed and stressed groups were 3.448mm and 3.461mm, respectively, while there was no attachment loss in the control group. A statistically significant difference was found among these groups (P < 0.001), as illustrated in (Table 2).

The present study showed a significant positive association between periodontal parameters and salivary RANKL levels among groups, as illustrated in (Table 3).

**Table 3:** The Spearman Correlation of the Periodontal Parameters and Salivary RANKL Levels Among Groups

Periodontal Parameters	RANKL (ng/L)	
	Correlation	P-value
Age (years)	0.310	0.003**
PLI (%)	0.651	0.000***
BOP (%)	0.679	0.000***
PPD (mm)	0.711	0.000***
CAL (mm)	0.718	0.000***
Stress	0.580	0.000***

**Notes**: Significance level **P≤ 0.01, ***P ≤ 0.001,

**Abbreviations**: “PLI, plaque index; BOP, bleeding on probing; PPD, probing pocket depth, CAL, clinical attachment loss; RANKL, Receptor Activator Nuclear Factor KB ligand ”; %, Percent; mm, Millimeter; ng/L, Nanogram per liter.

## Discussion

The global incidence of occupational stress, which harms employers’ social psychology, is high.^22^ This study is the first to examine if occupational stress factors affect periodontal variables of periodontitis and compare it to healthy controls according to the salivary RANKL level in 90 Iraqi employees aged 30 to 50. Other authors worldwide conducted other studies using various target communities, indices, and scores to determine the relationship.^19^,^24^,^31^,^32^ In addition, studies measured psychological variables using various self-report scales “Minnesota Multiphasic Personality Inventory, Modifiers and Perceived Stress Scale, Brief Symptom Inventory, as well as stress, anxiety, and depression”.^33–35^ These variations may restrict investigation comparisons.

This study measured participants’ oral hygiene by the percentage of surfaces with plaque after disclosure. A total mean plaque score is slightly higher, with about (69) % of employees feeling occupational stress compared to employees without such stress. Furthermore, BOP was found in over 50% of sites, consistent with other studies’ results. More gingival inflammation among employees with occupational stress than those without stress might explain the increased BOP site percentage. These findings suggest that the impact of occupational stress on the parameters of periodontitis, which can be explained by unhealthy habits like poor oral hygiene and malnutrition, can drastically change oral health.^36–38^ Concurrently, occupational stress can lead to immune suppression and aggravating chronic inflammatory diseases like periodontitis.^39^ Findings uncorroborated this in a previous study, which exacerbated experimental gingivitis in a randomized split-mouth experiment and measured stress differently. The study found no association between stress, plaque accumulation, and gingival inflammation.^40^

Throughout our study, employees with occupational stress had a higher mean PPD and CAL, indicating a greater severity of periodontitis. Previous studies have found similar results for comparable groups. Occupational stress can be caused by bad working environments, workload over or under, long hours (more than 40 hours per week), role in the organization, career advancement, work relationships, and work environment. Stress in employees may depend on social support or how an individual manages occupational stress because everybody experiences it differently.^29^

Several studies show workers inhibit good emotions, leading to increased psychosocial stress and lower occupational enjoyment. In some workers, overwork-related stress was connected to systemic diseases like cardiovascular attacks, myocardial infarction, diabetes, and hypertension.^41–43^ Our study added to the evidence suggesting that occupational stress possibly influences the presence of periodontitis and may be a risk indicator of periodontitis development; this finding agrees with several studies using a wide range of questionnaires and clinical measures have found that people with periodontal disease were more likely to report feeling stressed than healthy controls.^19^,^35^,^44^,^45^

The employees in the periodontitis with occupational stress group had significantly higher levels of salivary RANKL as an immunological mediator than those in the other groups; this proposed synergetic association between the elevated RANKL by stress and periodontal parameters, and thus an association between occupational stress and periodontal conditions.^46^,^47^ These results agree with other studies, which found increased RANKL concentrations in people with stress compared to healthy controls.^12^,^28^ Furthermore, a case–control study conducted by Mengel et al searched for the correlation between different levels of immunological mediators and glucocorticoids and subjective measures of stress. Overall, no statistically significant difference was found in stress levels between the controls and cases and the levels of inflammatory mediators and glucocorticoids had no positive correlation with stress recorded via the questionnaire.^48^

Some limitations of the study were identified. At first, no other possible confounders leading to periodontal disease, such as poor health knowledge, low socioeconomic status, and education level, were examined. An additional limitation is that the study’s observational design may have revealed stress and periodontal disease temporal relationships. Thus, longitudinal studies of the sequence of risk indicators of periodontitis beginning and progressing are needed to define causing links between possibility stressors and periodontitis.

## Conclusion

The present study findings suggest that occupational stress may predispose indicators to increased risk levels of periodontitis among a group of Iraqi employees. Therefore, occupational stress should also be considered when managing periodontitis among employees.
